# Prevalence of intestinal opportunistic parasites infections in the University hospital of Bobo-Dioulasso, Burkina Faso

**DOI:** 10.1186/s40249-015-0065-x

**Published:** 2015-07-27

**Authors:** Ibrahim Sangaré, Sanata Bamba, Mamoudou Cissé, Adama Zida, Rabila Bamogo, Constant Sirima, Bienvenue K. Yaméogo, Roger Sanou, François Drabo, Roch K. Dabiré, Robert T. Guiguemdé

**Affiliations:** Institut Supérieur des Sciences de la Santé, Université Polytechnique de Bobo-Dioulasso, 01 BP 1091 Bobo-Dioulasso, Burkina Faso; Centre MURAZ, Unité de Recherche en Paludisme et Maladies Tropicales Négligées, Bobo-Dioulasso, Burkina Faso; Centre Hospitalier Universitaire Souro Sanou, Bobo-Dioulasso, Burkina Faso; Centre Hospitalier Universitaire Yalgado Ouédraogo, Ouagadougou, Burkina Faso; Institut de Recherche en Sciences de la Santé, Direction Régionale de l’ouest, Ouagadougou, Burkina Faso; Ministère de la Santé, Direction de la Lutte contre la Maladie, Coordination de la lutte contre les Maladies tropicales Négligées, Ouagadougou, Burkina Faso

**Keywords:** Intestinal opportunistic parasite, Prevalence, Bobo-Dioulasso, Burkina Faso

## Abstract

**Background:**

Gastrointestinal parasites infections are widespread in Africa and their prevalence infections vary from country to country. This study aimed at assessing the prevalence of opportunistic intestinal parasites infection and other gastrointestinal parasites infection among patients attending the laboratory of Parasitology and Mycology of the University Hospital Souro Sanou of Bobo-Dioulasso.

**Methods:**

A hospital cross-sectional based study was conducted from April to August, 2012. Participants were persons whom parasitological examination of stools has been prescribed by a clinician. The stools examination methods included direct wet saline examination, lugol’s iodine staining technique, formol-ether concentration and modified Ziehl-Neelsen staining. We recorded age and sex information for each patient.

**Results:**

The overall prevalence of intestinal parasite infections was 65.3 % (190/291). Majority of the parasitic infections was waterborne (64.3 %) consisting of high prevalence of *Cryptosporidium sp.* (26.5 %) and *Entamoeba histolytica/dispar* (23.4 %). The prevalence of opportunistic parasites was 28.9 % and *Cryptosporidium sp*. was the most prevalent species followed by *Blastocystis sp*. (1.0 %), *Cyclospora sp*. (0.7 %) and *Isospora belli* (0.7 %). The prevalence of intestinal helminthes was 1.7 %.

**Conclusions:**

The prevalence of intestinal parasitism in general remains high in Bobo-Dioulasso requiring the establishment of adequate diagnostic techniques, treatment and prevention.

## Background

Intestinal parasitic infections caused by helminthes and protozoans are among the most widespread and remain an important cause of morbidity and mortality in developing countries [1]. The prevalence of infection is remarkably high in sub-Saharan Africa, where the largest burden of human immunodeficiency virus/ acquired immunodeficiency syndrome (HIV/AIDS) cases is concentrated [2, 3]. HIV infections, tropical and subtropical climate, high population density, poverty, very low hygienic conditions and health education are the major factors for the transmission of intestinal parasites [1].

Indeed in the previous studies, the prevalence of certain intestinal parasite known as opportunistic is significantly higher among HIV infected individuals with chronic diarrhea and CD4 lymphocytes counts of 200 cells/mm^3^ [4–8]. The presence of the opportunistic intestinal parasites, *Cryptosporidium sp.*, *Cyclospora cayetanensis*, *Isospora belli*, *Blastocytis hominis* and *Microsporidia* is well documented in patients with HIV/AIDS [4, 5, 9–12]. However, the incidence and the prevalence of the opportunistic intestinal parasites in HIV/AIDS patients are likely to depend upon the endemicity in the community.

Infections by the major human gastrointestinal parasites are widespread in Africa and the prevalence of these infections varies from country to country. Intestinal parasites are a major public health problem in Burkina Faso [13]. Their prevalence was estimated at 54.7 % throughout the country [14]. In addition, the epidemiology of intestinal parasitism is very wide and is significantly different between sahelian area (38.9 % in the Sahel) and humid area (65.8 % in the East) [13]. In the capital city of Ouagadougou, a prevalence of 60.82 and 52.47 % have been previously reported respectively by Ouermi *et al*., [15] and Karou *et al*., [16]. In Bobo-Dioulasso, economic capital of the country, the prevalence of intestinal parasites was 23.8 % [17]. Published data about opportunistic intestinal parasites infection in Burkina Faso are extremely rare. Only one study performed in 1998 at the Pediatric Department of Bobo-Dioulasso Hospital showed a prevalence of 5.2 % of cryptosporidiosis [18].

This study aimed at assessing the prevalence of opportunistic intestinal parasites among gastrointestinal parasites in patients attending the Laboratory of Parasitology and Mycology of the University Hospital Souro Sanou of Bobo-Dioulasso for diagnosis.

## Methods

### Study design

A descriptive cross-sectional hospital based study was carried out from April to August, 2012 in the University Hospital Souro Sanou of Bobo-Dioulasso, Burkina Faso.

Bobo-Dioulasso (11°10′42″N; 4°17′35″W) is located in the western part of the country at 360 km from Ouagadougou, the capital city of Burkina Faso. The city population is around 1.5 million inhabitants. The climate is Sudan climatic types and is characterized by a rainy season from June to October with relatively abundant rainfalls (the annual rainfall ranges from 1000 to 1200 mm) and a dry season from November to May. The annual average temperature is about 28 °C. Sanitary conditions are insufficient in large sectors of the city.

The University Hospital Souro Sanou is the only referral and teaching hospital in the region. The hospital provides various health services.

Participants were persons whom parasitological examination of stools has been prescribed by a clinician. The sample size was determined using the single proportion population formula. It was calculated assuming a prevalence of 50 % with a margin of error of 0.05 and a confidence level of 95 %, that a minimum size of 291 participants.

### Fecal sample collection

A written informed consent form has been administered to each participant. Then, a single fresh stool sample was collected from each consenting study participant in a sterile fecal container early morning. The specimens were soon transported to the laboratory of Parasitology-Mycology of the University Hospital Souro Sanou of Bobo-Dioulasso.

Sociodemographic characteristics of the study participants including age and sex were registered for each patient on the data collection form.

### Parasitological methods

Freshly stool specimens were collected, processed and examined microscopically in saline wet mount to detect larva, eggs, trophozoites and cysts of various parasites. In addition, formol-ether concentration was performed and modified Ziehl-Neelsen (ZN) method was used to detect coccidian species.

### Direct examination

A portion of stool was examined by direct wet saline mount preparation (0.90 % sodium chloride solution) to observe motile intestinal parasites and trophozoites under light microscope at 10× and 40× magnifications. Lugol’s iodine staining method was also performed to observe cysts of the intestinal protozoan parasites.

The remaining part of stool was processed by the following methods.

### Formalin ether concentration

About 7 ml of 10 % formalin was added to approximately 1 g of feces and mixed using an applicator stick. The stool sample was sieved with cotton gauze and transferred to 15 ml centrifuge tube Falcon®. After adding 3 ml of diethyl ether to the mixture and hand shaking, the content was centrifuged at 2000 rpm for 3 min. The supernatant was poured and a drop of sediment was transferred to slide. Finally, the entire zone under the cover slip was systematically examined using 10X and 40X objective lenses to observe ova, cyst and larvae of different intestinal parasites according to the protocol of Ritchie [19, 20].

### Modified Ziehl-Neelsen method

In this method, thin smears were prepared from preserved as well as sediments of concentrated stool samples, air-dried, and fixed with absolute methanol for 5 min. The smears were stained with carbol-fuchsin for 10 min and thereafter, washed with tap water. The slides were decolorized in chlorhydric acid-ethanol 1 % for 2 min and were counter stained with methlyene blue 0.25 % for 1 min [21, 22]. Finally the stained smears were examined using oil immersion objective to detect oocysts of *Cryptosporidium* sp., of *Isospora belli*, of *Cyclospora sp.* and cyst of *Blastocystis sp.*

### Quality control

Each stool sample was examined by two different laboratory technicians. In case of discordant results, the stool sample slide was read by a third technician, and his report was considered as the final result.

Randomly selected samples were also sent to the laboratory of Parasitology of Centre MURAZ, Bobo-Dioulasso to check the reproducibility of the results.

### Data analysis

Data were double-entered based on EpiData 3.1. Statistical analysis was performed with SPSS Statistics 17.0 (SPSS Inc., Chicago, IL). Chi-square test was used to compare the categorical variables. Fisher’s exact test was used when the expected value in any cell was less than 5.

### Ethics statement

The study protocol was approved by the institutional review boards of the University Hospital of Bobo-Dioulasso for routine analyses, Burkina Faso. Participants were contacted through the hospital practitioners and the objectives, procedures, and potential risks were carefully explained to all potential participants. Interested individuals were asked to sign a written inform consent or their parents in the case of minors before being involved in the study. Personal data form participant and all diagnostic results were kept strictly confidential. Results of participants with parasitic infections were sent, as soon as possible to clinicians for their case management.

## Results

### Participants’ characteristics

Among the 291 participants, 146 (50.2 %) were male, 145 (49.8 %) were female (Table 1) with a 1.1 sex ratio. The mean age of the participants was 29.2 ± 20.8 years (ranged, 0–89 years).

**Table 1:** Distribution of sociodemographic characteristics

	Total number of individual		
Age group (years)	Gender		Total
	Male	Female	
0–5	22	18	40 (13.7 %)
6–9	14	16	30 (10.3 %)
10–14	12	7	19 (6.5 %)
15–29	33	37	70 (24.1 %)
30–59	61	63	124 (42.6 %)
≥60	4	4	8 (2.7 %)
Total	146	145	291

### Prevalence of gastrointestinal parasite

The overall prevalence of intestinal parasite infections was 65.3 % (190/291). Majority of the parasitic infections were waterborne protozoa, prevalence of 64.3 % (187/291) with few helminthes, 1.7 % (5/291). *Cryptosporidium sp*. and *Entamoeba histolytica/dispar* were found at 26.5 and 23.4 %, respectively, constituting the majority of parasitic infections, followed by others protozoa *Entamoeba coli* (19.6 %), *Giardia lamblia* (4.8 %), *Trichomonas intestinalis* (1.7 %), *Blastocystis hominis* (1.0 %), *Cyclospora sp.* (0.7 %) and *Isospora belli* (0.7 %) were also observed (Table 2). Among the helminthic parasites, one case of eggs of *Ascaris lumbricoides*, *Trichuris trichiura*, *Ankylostomidae*, *Hymenolepis nana*, *Dicrocoelium sp* and larvae of *Strongyloides stercoralis* were observed at a rate of 0.3 % respectively.

**Table 2:** Prevalence of different species intestinal parasites

Parasite species	Number of positive samples (Prevalence %)
Protozoa	187 (64.3)
*Entamoeba histolytica*	68 (23.4)
*Entamoeba coli*	57 (19.6)
*Giardia lamblia*	14 (4.8)
*Trichomonas intestinalis*	5 (1.7)
*Cryptosporidium sp.*	77 (26.5)
*Blastocystis sp*	3 (1.0)
*Cyclospora*	2 (0.7)
*Isospora belli*	1 (0.3)
Helminth	5 (1.7)
*Ankylostomidae*	1 (0.3)
*Ascaris lumbricoides*	1 (0.3)
*Dicrocoelium sp*	1 (0.3)
*Hymenolepis nana*	1 (0.3)
*Trichuris trichiura*	1 (0.3)

### Prevalence of intestinal opportunistic parasites

Regarding the intestinal opportunistic parasites infection, the results revealed a total of 84 out the 291 patients, thus the prevalence was 28.9 %. *Cryptosporidium sp*. infection was the most diagnosed (26.5 %, 77/291) followed by infections with *Blastocystis sp*. (1.0 %; 3/291), *Cyclospora* (0.7 %, 2/291) and *Isospora belli* (0.7 %, 2/291) (Table 3).

**Table 3:** Prevalence of intestinal opportunistic intestinal parasites according age and sex

	Opportunistic parasite			*Cryptosporidium*	
Risk factor	No. samples examined	No. positive (Prevalence %)	P value	No. positive (Prevalence %)	P value
Age group (years)					
0–5	40	18 (45)		15 (37.5)	
6–9	30	10 (33.3)		10 (33.3)	
10–14	19	5 (26.3)	0.2	5 (26.3)	0.5
15–29	70	16 (22.9)		16 (22.9)	
30–59	124	32 (25.8)		29 (23.4)	
>60	8	3 (37.5)		2 (25.0)	
Gender					
Male	146	44 (30,1)		41 (28.1)	
Female	145	40 (30,1)	0.6	36 (24.8)	0.5

A break-up of the 291 persons (146 males and 145 females) revealed no difference as both the sexes were equally affected with the opportunistic intestinal parasites. Both age (*P* = 0.6) was not significantly associated the opportunistic intestinal parasites (Table 3).

### Global co-infection of gastrointestinal parasite and prevalence co-infection of opportunistic parasites species

The overall prevalence of multiple infection of gastrointestinal parasite was 21.0 % (61/291) and co-infection of opportunistic parasites was 2.1 (6/291).

## Discussion

Overall 190 out of 291 (65.3 %) participants harbored intestinal parasites in the the laboratory of Parasitology and Mycology laboratory of University hospital of Bobo-Dioulasso. The national prevalence for intestinal parasite infection was 54.7 % [23].

This prevalence of intestinal parasitism is high compared with previous findings in Burkina Faso. Other authors reported 23.8 % in Bobo-Dioulasso [17, 23] 52.4 and 60.8 % in Ouagadougou [15, 16]. The difference could be due mainly to coproparasitological techniques used. In our study, modified Ziehl-Neelsen method was systematically performed. This technique increased the sensitivity of study by diagnosing 78 cases of parasitism compared to direct examination and formol ether concentration.

The high prevalence of protozoa parasitism, (64.3 %) compared to that of helminth infections (1.7 %) in this study is in agreement with previous findings in Burkina Faso [23] and in other developing countries in Africa [6, 24–26]. The drug mass administration with albendazole could explain the low rate of helminthes infection as previously reported [23, 27, 28].

The prevalence of intestinal opportunistic parasites was 28.9 % and the most prevalent species was *Cryptosporidium sp.* (26.5 %). This prevalence of *Cryptosporidium sp.* is higher than those reported in Burkina Faso (5.2 %) [18], and as well as in west Africa (ranging from 7.7 to 25.71 %) [29–31]. The difference could be due to sample size as our study presented a larger sample size than that reported by other authors.

The presence of opportunistic intestinal parasites such as *Blastocystis* (1.10 %), *Cyclospora* (0.7 %) and *Isospora belli* (0.7 %) should not be neglected. The pathogenicity of certain species such as *Blastocystis sp*. among immunocompetent persons is not excluded [32, 33].

Gender and age did not significantly affect the prevalence of intestinal parasitic infections while it was not a risk factor for acquiring these infections. The distribution of the parasitic infections in both sexes as well as, among the various age groups suggested that sex and age were not predetermining factors for parasitic infections in our study.

Our study had some limitations. First, host immunity such as HIV status was not investigated in the present study. Second, we did not use the Weber trichome staining or PCR which would have improved the diagnosis of microsporidiosis [34]. The present study is a pilot study and we are planning a large-scale longitudinal study.

One perspective of our staff is to use the molecular tool to study the epidemiology of different opportunistic species. In fact, for example, the identification and characterization of *Cryptosporidium* species and population variants (genotypes and subgenotypes) is fundamental to study the epidemiology of cryptosporidiosis, being a valid support for prevention and control strategies [35]. The large scale of human cases of cryptosporidiosis worldwide are caused by two species, *Cryptosporidium parvum* and *C. hominis* [36]. However, other species, including *C. felis*, *C. meleagridis*, *C. canis, C. suis, C. muris* and, more rarely, *C. cuniculus*, *C. ubiquitum* and *C. andersoni* can also infect humans, especially children under the age of 5 years and immunocompromised individuals [37, 38]. Oocyst morphology, host specificity or preferences in infection sites do not provide sufficient information for the identification of Cryptosporidium species, genotypes or subgenotypes. The confirmation of their species status and determination of virulence and pathogenic profiles might explain why some patients are asymptomatic while others present clinical symptoms [38].

## Conclusions

The overall prevalence of intestinal parasites was therefore 65.3 % in our study. The prevalence of intestinal opportunistic parasites was 28.9 % and *Cryptosporidium sp*. was the most prevalent species.

Prompt diagnosis of parasitic infections in HIV-negative and positive patients, especially intestinal parasitic infections using staining and molecular diagnostic tools is advocated in order to improve the management and quality of life of HIV-infected individuals.

## Abbreviations

CI: Confident interval
°C: Degree celsius
CD4: Lymphocyte cluster differentiation 4
HIV/AIDS: Human immunodeficiency virus/ acquired immunodeficiency syndrom
%: Percent
mm: Millimeter
ZN: Ziehl-Neelsen

## References

[1] Harhay M, Horton J, Olliaro P (2010). Epidemiology and control of human gastrointestinal parasites in children. Expert Rev Anti Infect Ther.

[2] Bhutta Z, Sommerfeld J, Lassi Z, Salam R, Das J (2014). Global burden, distribution, and interventions for infectious diseases of poverty. Infect Dis Poverty.

[3] Murray C, Ortblad K, Guinovart C, Lim S, Wolock T, Roberts D (2013). Global, regional and national incidence and mortality for HIV, tuberculosis, and malaria during 1990–2013: a systematic analysis for the Global Burden of Disease Study 2013. Lancet.

[4] Adamu H, Petros B, Zhang G, Kassa H, Amer S, Ye J (2014). Distribution and clinical manifestations of Cryptosporidium species and subtypes in HIV/AIDS patients in Ethiopia. PLoS Negl Trop Dis.

[5] Adamu H, Wegayehu T, Petros B (2013). High prevalence of diarrhoegenic intestinal parasite infections among non-ART HIV patients in Fitche Hospital, Ethiopia. PLoS One.

[6] Vouking M, Enoka P, Tamo C, Tadenfok C (2014). Prevalence of intestinal parasites among HIV patients at the Yaounde Central Hospital, Cameroon. Pan Afr Med J.

[7] Wanyiri J, Kanyi H, Maina S, Wang D, Steen A, Ngugi P (2014). Cryptosporidiosis in HIV/AIDS patients in Kenya: clinical features, epidemiology, molecular characterization and antibody responses. Am J Trop Med Hyg.

[8] Feasey N, Healey P, Gordon M (2011). Review article: the aetiology, investigation and management of diarrhoea in the HIV-positive patient. Aliment Pharmacol Ther.

[9] Wegayehu T, Adamu H, Petros B (2013). Prevalence of Giardia duodenalis and Cryptosporidium species infections among children and cattle in North Shewa Zone, Ethiopia. BMC Infect Dis.

[10] Calvo M, Carazo M, Arias M, Chaves C, Monge R, Chinchilla M (2004). Prevalence of Cyclospora sp., Cryptosporidium sp, microsporidia and fecal coliform determination in fresh fruit and vegetables consumed in Costa Rica. Arch Latinoam Nutr.

[11] Wumba R, Jean M, Benjamin L, Madone M, Fabien K, Josue Z (2012). Enterocytozoon bieneusi identification using real-time polymerase chain reaction and restriction fragment length polymorphism in HIV-Infected Humans from Kinshasa Province of the Democratic Republic of Congo. J Parasitol Res.

[12] Wumba R, Longo-Mbenza B, Menotti J, Mandina M, Kintoki F, Situakibanza N (2012). Epidemiology, clinical, immune, and molecular profiles of microsporidiosis and cryptosporidiosis among HIV/AIDS patients. Int J Gen Med.

[13] Fortunato S, Castagna B, Monteleone M, Pierro R, Cringoli G, Bruschi F (2014). Parasite prevalence in a village in Burkina Faso: the contribution of new techniques. J Infect Dev Ctries.

[14] Cisse M, Coulibaly S, Guiguemde R (2011). Epidemiological features of intestinal parasitic infection in Burkina Faso from 1997 to 2007. Med Trop (Mars).

[15] Ouermi D, Karou D, Ouattara I, Gnoula C, Pietra V, Moret R (2012). Prevalence of intestinal parasites at Saint-Camille medical center in Ouagadougou (Burkina Faso), 1991 to 2010. Med Sante Trop.

[16] Karou S, Sanou D, Ouermi D, Pignatelli S, Pietra V, Moret R (2011). Enteric parasites prevalence at Saint Camille Medical Centre in Ouagadougou, Burkina Faso. Asian Pac J Trop Med.

[17] Sangare I, Zida A, Bamba S, Cissé M, Coulibaly S, Guiguemde R (2013). Bilan des demandes d’examens parasitologiques des selles et des parasitoses diagnostiquées de 1999 à 2008 au laboratoire de Parasitologie du Centre Muraz, Bobo-Dioulasso, Burkina Faso. Annales de l’Université de Ouagadougou-Série D.

[18] Nacro B, Bonkoungou P, Nagalo K, Tall F, Curtis V (1998). Clinical profile of cryptosporidiosis in a pediatric hospital environment in Burkina Faso. Med Trop (Mars).

[19] Ritchie L (1948). An ether sedimentation technique for routine stool examinations. Bull U S Army Med Dep.

[20] Lindo J, Levy V, Baum M, Palmer C (1998). Epidemiology of giardiasis and cryptosporidiosis in Jamaica. Am J Trop Med Hyg.

[21] Clarke S, McIntyre M (1996). Modified detergent Ziehl-Neelsen technique for the staining of Cyclospora cayetanensis. J Clin Pathol.

[22] Henriksen S, Pohlenz J (1981). Staining of cryptosporidia by a modified Ziehl-Neelsen technique. Acta Vet Scand.

[23] Cissé M, Bamba S, Zida A, Sangare I, Guiguemdé R (2011). Prevalence de l’ankylostommiase avant et après la mise en oeuvre du traitement de masse à l’ivermectine et à l’albendazole au Burkina Faso. Science et Technique Science de la santé.

[24] Mwakitalu M, Malecela M, Mosha F, Simonsen P (2014). Urban schistosomiasis and soil transmitted helminthiases in young school children in Dar es Salaam and Tanga, Tanzania, after a decade of anthelminthic intervention. Acta Trop.

[25] Ndiaye D, Ndiaye M, Gueye P, Badiane A, Fall I, Ndiaye Y (2013). Intestinal helminthiasis diagnosed in Dakar, Senegal. Med Sante Trop.

[26] Nwaneri D, Omuemu V (2013). Prevalence and intensity of intestinal helminthiasis in children living in orphanages in Benin City, Nigeria. J Prev Med Hyg.

[27] Belizario V, Liwanag H, Naig J, Chua P, Madamba MI, Dahildahil R (2014). Parasitological and nutritional status of school-age and preschool-age children in four villages in Southern Leyte, Philippines: Lessons for monitoring the outcome of Community-Led Total Sanitation. Acta Trop.

[28] Supali T, Djuardi Y, Bradley M, Noordin R, Ruckert P, Fischer P (2013). Impact of six rounds of mass drug administration on Brugian filariasis and soil-transmitted helminth infections in eastern Indonesia. PLoS Negl Trop Dis.

[29] Ka R, Dia N, Dia M, Tine D, Diagne R, Diop S (2011). Parasitic and bacterial etiologies of diarrhea among people living with HIV hospitalized in Fann hospital (Senegal). Mali Med.

[30] Minta D, Dembele M, Dolo A, Sidibe A, Diarra A, Konate A (2007). Digestive parasitic diseases to HIV/AIDS infected patients of internal medicine and infectious diseases wards of the hopital du Point “G” Bamako - Mali. Mali Med.

[31] Kassi R, Kouassi R, Yavo W, Barro-Kiki C, Bamba A, Menan H (2004). Cryptosporidiosis and isosporiasis in children suffering from diarrhoea in Abidjan. Bull Soc Pathol Exot.

[32] Chandramathi S, Suresh K, Sivanandam S, Kuppusamy U (2014). Stress exacerbates infectivity and pathogenicity of Blastocystis hominis: in vitro and in vivo evidences. PLoS One.

[33] Santos H, Sodre F, de Macedo H (2014). Blastocystis sp. in splenic cysts: causative agent or accidental association? A unique case report. Parasit Vectors.

[34] De A (2013). Current laboratory diagnosis of opportunistic enteric parasites in human immunodeficiency virus-infected patients. Trop Parasitol.

[35] Putignani L, Menichella D. Global distribution, public health and clinical impact of the protozoan pathogen cryptosporidium. Interdiscip Perspect Infect Dis. 2010; 2010.doi: 10.1155/2010/753512. 10.1155/2010/753512 PMC2913630 20706669

[36] Gasser R, Zhu X, Caccio S, Chalmers R, Widmer G, Morgan U (2001). Genotyping Cryptosporidium parvum by single-strand conformation polymorphism analysis of ribosomal and heat shock gene regions. Electrophoresis.

[37] Checkley W, White A, Jaganath D, Arrowood M, Chalmers R, Chen X (2014). A review of the global burden, novel diagnostics, therapeutics, and vaccine targets for cryptosporidium. Lancet Infect Dis.

[38] Jiang Y, Ren J, Yuan Z, Liu A, Zhao H, Liu H (2014). Cryptosporidium andersoni as a novel predominant Cryptosporidium species in outpatients with diarrhea in Jiangsu Province, China. BMC Infect Dis.

